# Genetic weighted k-means algorithm for clustering large-scale gene expression data

**DOI:** 10.1186/1471-2105-9-S6-S12

**Published:** 2008-05-28

**Authors:** Fang-Xiang Wu

**Affiliations:** 1 Department of Mechanical Engineering, University of Saskatchewan, Saskatoon, SK, S7N 5A9, Canada; 2 Division of Biomedical Engineering, University of Saskatchewan, Saskatoon, SK, S7N 5A9, Canada

## Abstract

**Background:**

The traditional (unweighted) k-means is one of the most popular clustering methods for analyzing gene expression data. However, it suffers three major shortcomings. It is sensitive to initial partitions, its result is prone to the local minima, and it is only applicable to data with spherical-shape clusters. The last shortcoming means that we must assume that gene expression data at the different conditions follow the independent distribution with the same variances. Nevertheless, this assumption is not true in practice.

**Results:**

In this paper, we propose a genetic weighted K-means algorithm (denoted by GWKMA), which solves the first two problems and partially remedies the third one. GWKMA is a hybridization of a genetic algorithm (GA) and a weighted K-means algorithm (WKMA). In GWKMA, each individual is encoded by a partitioning table which uniquely determines a clustering, and three genetic operators (selection, crossover, mutation) and a WKM operator derived from WKMA are employed. The superiority of the GWKMA over the k-means is illustrated on a synthetic and two real-life gene expression datasets.

**Conclusion:**

The proposed algorithm has general application to clustering large-scale biological data such as gene expression data and peptide mass spectral data.

## Background

Clustering is defined as a process of partitioning a set of objects (patterns) into a set of disjoined groups (clusters). Its goal is to reduce the amount of data by categorizing or grouping similar data items together and obtain useful information. Clustering methods can be divided into two basic types: hierarchical and partitional clustering [1]. Within each type there exists a wealth of subtypes and different algorithms. Hierarchical clustering proceeds successively either by merging smaller clusters into larger ones (bottom-up), or by splitting larger clusters into smaller clusters (top-down). The hierarchical clustering methods differ in the rules used to decide which two small clusters are merged or which large cluster is split. The final result of the algorithm is a binary tree of clusters called a dendrogram, which shows how the clusters are related to each other. By cutting the dendrogram at a desired level, a clustering of objects in a dataset into disjoint groups is obtained.

On the other hand, partitional clustering – k-means, for example – attempts to directly divide a dataset into a number of disjoint groups. All partitional clustering algorithms need as input the number of clusters and a cost (criterion) function to define the quality of a partition. The partitional clustering method aims at optimizing the cost function to minimize the dissimilarity of the objects within each cluster, while maximizing the dissimilarity of different clusters. In general, the partitional clustering algorithms are iterative and hill-climbing, and thus they are sensitive to the choice of the initial partition. Furthermore, since the associated cost functions are nonlinear and multimodal, usually these algorithms converge to a local minimum. The algorithms based on combinatorial optimization such as integer programming, dynamic programming and, branch-bound methods are too expensive since the number of partitions of *n *objects into *k *clusters is *O*(*k*^*n*^).

Genetic algorithms (GA) [2], inspired by natural evolution of genes, offer heuristic solutions to some optimization problems. The algorithm typically starts with a set of solutions (randomly generated) called the population and creates successive, new generations of the population by genetic operations such as natural selection, crossover, and mutation. Natural selection is performed based on the fitness (related to the cost function) of an individual. For an individual, the better its fitness, the more chances it has to survive in the next generation. Crossover is performed by certain crossover rule and mutation aims at changing an individual by a user-specified mutation probility. The intuition underlying the approach is that each new population will be better than the previous one. Actually it has been proved [3] that a canonical GA converges to the global optimum with probability 1.

A GA is highly dependent on the coding of the solutions (individuals). In the context of weighted k-means, a natural representation of a solution is a pair of variables (partitional string, cluster centroids). The partitional string describes for each object the index of cluster which it belongs to. The cluster centroids are representative objects of the clusters and their attributes are found by averaging the corresponding attributes among the objects in a particular cluster. These two variables depend on each other such that if one of them is given, the other one can be uniquely constructed. Since the cluster centroids generally are real numbers, it might be very difficult to encode them. On the other hand, a direct encoding for partitional strings is a simple problem.

Genetic algorithms have been previously considered for clustering problems [4-11]. Often genetic algorithms are not hybridized with k-means algorithms [5,6,9,11] and thus their rates of convergence were very slow. On the other hand, when GA are hybridized with k-means algorithms [7,8,10], the resultant algorithms inherit some drawbacks of unweighted k-means algorithms, for example, that the resultant clusters are spherical-shape. Further, if the inherent structure of the clusters in the data is not spherical shaped, such algorithms can not give the correct results

In this paper, we propose a genetic weighted k-means algorithm (GWKMA). This is a hybrid approach to combining a GA with the weighted k-means algorithm (WKMA) [12,13] and partially remedies drawbacks of other attempts [4-11]. The GWKMA encode the solutions by partitional strings and employs three genetic operations – natural selection, crossover and mutation – and one WKM operation derived from the weighted k-means algorithm (WKMA).

## Methods

### WKMA

In a general sense, a *k*-partitioning algorithm takes as input a set D = {*x*_1_, *x*_2_⋯, *x*_*n*_} of *n *objects and an integer *K*, and outputs a partition of *D *into exactly *K *disjoint subsets *D*_1_,⋯, *D*_*K*_. Denote such a partition by Δ. Each of the subsets is a cluster, with objects in the same cluster being somehow more similar to each other than they are to all subjects in other different clusters. One way to make the determination of Δ into a well-defined problem is to define a cost function which measures the clustering quality of any partitions of a dataset.

In this paper, each attribute of an object (gene) is expressed as a real number and thus each object may be described by a real number row vector of dimension *d*, where *d *is the number of attributes of an object. Assume that all objects in the dataset have the same number of attributes, i.e. no missing data. Let (*x*_*i*_, *i *= 1,⋯, *n*) be a dataset of *n *objects. Let *x*_*ij *_denote the *j*th attribute of object *x*_*i*_. ***X ***= (*x*_*ij*_) is called an attribute matrix of object set *D*. For the predefined number *K *of clusters, the cost function for a weighted k-means clustering technique may be defined by

(1) $$J_{G}(\Delta )=\sum_{k=1}^{K}\sum_{x_{i}\in D_{k}}(x_{i}-{\bar{m}}_{k})G(x_{i}-{\bar{m}}_{k})'$$

where

(2) $${\bar{m}}_{k}=\frac{1}{n_{k}}\sum_{x_{i}\in D_{k}}x_{i}$$

*n*_*k *_and *m*_*k *_are the mean and the number of objects in *D*_*k*_, respectively, and *G *is a weighted matrix which is a symmetrical positive. The objective of a weighted k-means algorithm is to find an optimal partition expressed by Δ* and a symmetrical positive matrix G* satisfying equation (3) such that

(3) $$J_{G^{*}}(\Delta^{*})=\underset{\Delta }{\min}\{J_{G^{*}}(\Delta )\}$$

Obviously, given a partition Δ, the value of *J*_*G*_(Δ) change with the multiplication of a weighted matrix *G*. Therefore the weighted matrix must be normalized. In this study the determinant of *G *is set to be 1, i.e.

(4) (det(*G*)) = 1

For fixed *G *= *I *in equation (1), condition (4) is satisfied automatically, and equations (1) and (3) become the cost function and optimal objective of a traditional k-means algorithm, respectively.

For a fixed partition, we wish to determine *G *such that the cost function (1) is optimized under the normalization condition (4). To do that, we form the Lagrangian function

(5) $$L(G,\lambda )=\sum_{k=1}^{K}\sum_{x_{i}\in D_{k}}(x_{i}-{\bar{m}}_{k})G(x_{i}-{\bar{m}}_{k})'-\lambda (\det(G))-1)$$

and calculate its derivatives with respect to *G*

(6) $$\frac{\partial }{\partial G}L(G,\lambda )=\sum_{k=1}^{K}\sum_{x_{i}\in D_{k}}(x_{i}-{\bar{m}}_{k})'(x_{i}-{\bar{m}}_{k})-\lambda G^{-1}(\det(G))$$

Equating the derivative to zero and using the auxiliary condition (4) lead to

*W *= *λG*^-1^(det(*G*)) = *λG*^-1^

where $W=\sum_{k=1}^{K}W_{k}$ and $W_{k}=\sum_{x_{i}\in D_{k}}\left(x_{i}-{\bar{m}}_{k}\right)'(x_{i}-{\bar{m}}_{k})$ is the within-group variance of cluster *k *((*k *= 1,⋯, *K*), and

*λ *= (det(*W*))^1/*d*^

Finally, we have

(7) *G *= *W*^-1^(det(*W*))^1/*d*^

Note that *W *is dependent on partition Δ. To avoid ambiguousness, denote *W *induced by Δ as *W*(Δ). Substituting (7) into (1) leads to *J*(Δ) = *d*(det(*W*(Δ)))^1/*d*^. As *d *is a constant for a given dataset, the cost function of a weighted k-mean clustering is reduced to

(8) *J*(Δ_*o*_) = (det(*W*(Δ)))^1/*d*^

Thus the objective of a weighted k-mean algorithm is simplified as finding an optimal partition expressed by Δ_*o *_which minimizes

(9) $$J(\Delta_{o})=\underset{\Delta }{\min}{\left(\det(W(\Delta ))\right)}^{1/d}$$

There are *O*(*k*^*n*^) different partitions of *n *objects into exactly *k *clusters [1]. It is impractical to using an exhaustive search for the solution to clustering a large-size gene expression dataset. To overcome this problem, a heuristic approach is usually considered. The basic idea in the heuristic approach is to randomly select an initial partition and then move objects between groups if such moves make *J *significantly smaller.

Now consider how the cost function *J *changes when an object *x *currently in cluster *D*_*i *_is tentatively moved to a different cluster *D*_*j*_. Let Δ = (*D*_1_,⋯, *D*_*k*_), Δ' = (*D*_1_,⋯, *D*_*i*_\{*x*},⋯*D*_*k*_), and Δ" = (*D*_1_,⋯, *D*_*i*_\{*x*},⋯, *D*_*j *_∪ {*x*},⋯, *D*_*k*_) (*i *≠ *j*). Obviously the condition for successfully moving *x *from *D*_*i *_into *D*_*j *_is

(10) det(*W*(Δ")) < det(*W*(Δ))

From the definitions, it follows that

(11) $$W(\Delta )=W(\Delta ')+\frac{m_{i}}{m_{i}-1}(x-{\bar{x}}_{i})'(x-{\bar{x}}_{i})$$

(12) $$W(\Delta ")=W(\Delta ')+\frac{m_{j}}{m_{j}+1}(x-{\bar{x}}_{j})'(x-{\bar{x}}_{j})$$

Condition (10) is reduced to

(13) $$\frac{m_{j}}{m_{j}+1}(x-{\bar{x}}_{j}){\left[W(\Delta ')\right]}^{-1}(x-{\bar{x}}_{j})'<\frac{m_{i}}{m_{i}-1}(x-{\bar{x}}_{i}){\left[W(\Delta ')\right]}^{-1}(x-{\bar{x}}_{i})'$$

since det(*A *+ *βy'y*) = det(*A*)(1 + *βyA*^-1 ^*y'*) for any *d *× *d *invertible matrix *A*, any d – dimensional row vector *y*, and any number *β*.

**Figure 1:**
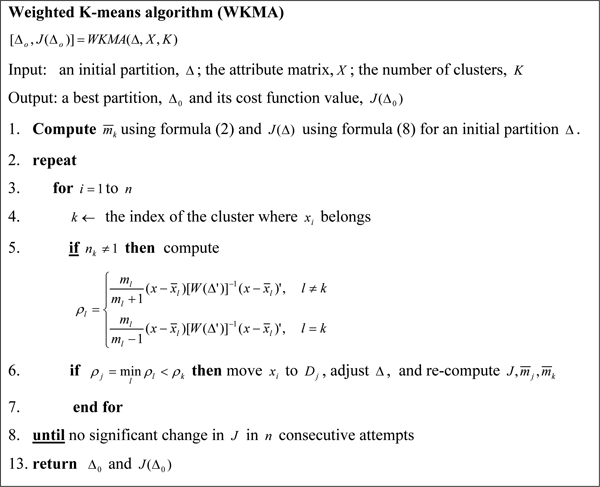
Iterative optimal k-means algorithm.

If reassignment is profitable, the greatest decrease in the cost function is obtained by selecting the cluster for which $\frac{m_{j}}{m_{j}+1}(x-{\bar{x}}_{j}){\left[W(\Delta ')\right]}^{-1}(x-{\bar{x}}_{j})'$ is minimal. This leads to the iteratively optimal weighted k-means algorithm (WKMA) shown in Figure 1.

### GWKMA

**Figure 2:**
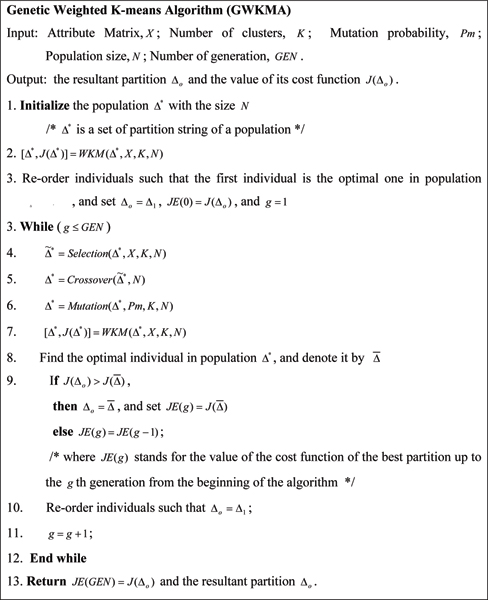
Genetic weighted k-means algorithm (GKMA).

As the WKMA is sensitive to initial partitions and its result is prone to the local minima, this paper proposes a genetic weighted k-means algorithm (GWKMA), shown in Figure 2. The GWKMA is a hybridization of GA and WKMA, including the three genetic operators in general GA and a WKM operator derived from WKMA. In the following we specify in details the encoding, selection, crossover, mutation, and WKM operators.

#### Encoding

In the literature [5,6,8], solutions (individuals) are encoded by the centers of clusters. Note that the centers of clusters are real numbers for general cluster tasks and the encoding of the real number in GA algorithms is hard and may degrade the accuracy of the solutions.

We use a partitional string to express a solution to a clustering. A partitional string is an integer string over the set {1,⋯, *K*}, on which each position corresponds to an object and the number in a position represents the cluster to which the corresponding object is assigned. Thus, the search space consists of all integer strings *s*_Δ _with length *n *over the set {1,⋯, *K*}. A population is expressed by a set of partitional strings representing its individuals (solutions), denoted by ${\tilde{\Delta }}^{*}$ or Δ*. One may set some additional conditions to refine the search space. For example, to avoid a singular clustering, one may impose the constraint that each element in the set {1,⋯, *K*} appears at least once in *s*_Δ_.

The advantage of encoding the centers of clusters is that the resultant clusters from GA clustering are convex. GWKMA encodes the solutions (individuals) by integer strings (partitional strings). This simplifies the encoding of GA and does not degrade the accuracy of the solutions. Since GWKMA includes the weighted k-means operator, the resultant clusters from GWKMA are still convex.

#### Selection operator

${\tilde{\Delta }}^{*}$ = *Selection*(Δ*, *X*, *K*, *N*). For convenience of the manipulation, GWKMA always assigns the best individual found over time in the population to individual 1 and copies it to the next population. Operator ${\tilde{\Delta }}^{*}$ = *Selection*(Δ*, *X*, *K*, *N*) selects (*N*-1)/2 individuals from the previous population according to the probability distribution given by

(14) Ps(sΔi)=F(sΔi)/∑i=1NF(sΔi)

where *N *(odd positive integer) is the number of individuals in a population, *s*_Δ*i *_is the partitional string of individual *i*, and F(*s*_Δ*i*_) represents the fitness value of individual *i *in the current population. Fitness here is defined as

(15) *F*(*S*_Δ_) = *TJ *- *J*(*s*_Δ_)

where *J*(*s*_Δ_) is calculated by (8), and *TJ *is calculated by the following formula

(16) $$TJ=\det(S)=\det(\sum_{x\in D}(x-\bar{m})'(x-\bar{m}))$$

and where $\bar{m}=\frac{1}{n}\sum_{x\in D}x$. It is evident that *TJ *≥ *J*(*s*_Δ_) for any *s*_Δ _in the problem. Note that there are (*N*-1)/2+1 individuals in ${\tilde{\Delta }}^{*}$.

#### Crossover operator

Δ* = *Crossover*(${\tilde{\Delta }}^{*}$, *N*). The intention of the crossover operation is to create new (and hopefully better) individuals from two selected parent individuals. In GWKMA, of two parent individuals, one always is the first individual that is the optimal individual found over time, and another is one of the selected (*N*-1)/2 individuals from the parent population other than the first individual. In this paper, the crossover operator adopts the single-point crossover method for simplicity. Note that after the crossover operation population Δ* has *N *individuals.

#### Mutation operator

Δ* = *Mutation*(Δ*, *Pm*, *K*, *N*). Each position in a coding string is randomly selected with a user-set mutation probability *Pm*, and the number in the selected position is uniformly randomly replaced by another integer from the set {1,⋯ *K*}. In other work [14], such a mutation depends on the distance of the corresponding object from the cluster centoids. Actually such a complex strategy is not necessary because the WKM operator will be used. To avoid any singular partition (containing an empty cluster), the mutation operator also randomly assigns one object to a cluster which is empty after all genetic operations.

#### WKM operator

[Δ*, *J*(Δ*)] = *WKM*(Δ*, *X*, *K*, *N*): The WKM operator is obtained by calling WKMA for each individual *s*_Δ _in population Δ*. It is sufficient to run the repeat loop in AKMA for several times. Note that *J*(Δ*) is an *N*-dimensional vector, each component of which corresponds to the value of the cost function of an individual in population Δ*. In other works [7-9], several different k-means operators were employed, and their functions are similar to that of WKMA. However, those k-means algorithms are neither iteratively optimal nor weighted.

### Evaluation

The term "evaluation of a clustering method" usually refers to the ability of a given method to recover true clusters in a dataset. There have been several attempts to evaluate a clustering method on theoretical grounds [14,15]. Since a clustering result can be considered as a partition of objects into a number of groups, for evaluating a clustering method it is necessary to define a measure of agreement between two partitions of the same dataset. In the clustering literature, measures of agreement between partitions are referred to as external indices. Several such indices have been described [15,16]. This paper adopts the adjusted Rand index (ARI).

Consider two partitions of *N *objects: the *r*-cluster partition *U *= {*u*_1_,⋯ *u*_*r*_} and the *s*-cluster partition *V *= {*v*_1_,⋯, *v*_*s*_}. One may construct a contingency table (Table 1), where entry *n*_*ij *_is the number of objects that are both in clusters *u*_*i *_and *v*_*j*_, *i *= 1,⋯, *r*, *j *= 1,⋯, *s*. Let $n_{i.}=\sum_{j=1}^{s}n_{ij}$ and $n_{.j}=\sum_{i=1}^{r}n_{ij}$ denote the sum of row *i*{*i *= 1,⋯, *r*}and the sum of column *j *(*j *= 1,⋯, *s*) in the contingency table, respectively, and let $Z=\sum_{i=1}^{r}\sum_{j=1}^{s}n_{ij}^{2}$ and $V=\left(\begin{array}{c} N\\ 2 \end{array}\right)=N(N-1)/2$ (the number of pairs of *N *objects). Based on the contingency matrix of two partitions, the ARI is defined as [16,17]:

**Table 1:** Contingency table for two partitions of *n *objects

	*v*_1_	*v*_2_		*v*_5_	Total
*u*_1_	*n*_11_	*n*_12_	⋯	*n*_1*s*_	*n*_1._
*u*_2_	*n*_21_	*n*_22_	⋯	*n*_2*s*_	*n*_2._
⋮	⋮	⋮		⋮	⋮
*u*_*r*_	*n*_*r*1_	*n*_*r*2_	⋯	*n*_*rs*_	*n*_*r*._

Total	*n*_.1_	*n*_.2_	⋯	*n*_.*s*_	*n*_.. _= *n*

(17) $$ARI=\frac{\sum_{i=1}^{r}\sum_{i=1}^{s}\left(\begin{array}{c} n_{ij}\\ 2 \end{array}\right)-\frac{1}{V}\sum_{i=1}^{r}\left(\begin{array}{c} n_{i.}\\ 2 \end{array}\right)\sum_{i=1}^{s}\left(\begin{array}{c} n_{.j}\\ 2 \end{array}\right)}{\frac{1}{2}\left[\sum_{i=1}^{r}\left(\begin{array}{c} n_{i.}\\ 2 \end{array}\right)+\sum_{i=1}^{s}\left(\begin{array}{c} n_{.j}\\ 2 \end{array}\right)\right]-\frac{1}{V}\sum_{i=1}^{r}\left(\begin{array}{c} n_{i.}\\ 2 \end{array}\right)\sum_{i=1}^{s}\left(\begin{array}{c} n_{.j}\\ 2 \end{array}\right)}$$

The ARI is an adjusted Rand index [18] in that its expected value is 1 when they matched perfect and 0 when the two partitions are selected at random. Accordingly, the large value of ARI indicates the two partitions are highly in agreement. To investigate the sensitivity of the partition clustering methods to initial partitions, the clustering method is run with numerous different initial partitions. Then the average ARI (AARI) of all pair-wise resultant clusterings is calculated. This AARI indicates the sensitivity of the clustering method to initial partitions. The larger the value of AARI, the more insensitive (better) the clustering method is to initial partitions.

To evaluate the quality of the clusters, we propose a measure of internal consistency based on the singular value decomposition (SVD) of each cluster. To define internal consistency, suppose we are given a partition of our *n *× *m *dataset *X *into *K *disjoint clusters, where *m *is the number of time points and *n *is the number of genes. For the *j*th cluster (*j *= 1,⋯, *K*) we have a matrix *X*_*j *_of microarray measurements, where the rows are genes and the columns are time points, so that *X*_*j *_is a *n*_*j *_× *m *matrix, where *n*_*j *_is the number of genes in the *j*th cluster. Using the SVD, we decompose $X_{j}=U_{j}S_{j}V_{j}^{T}$, where *U*_*j *_and *V*_*j *_are orthogonal matrices and *S*_*j *_is a diagonal matrix whose entries describe the importance of the columns of *U*_*j *_and *V*_*j*_. The matrix *X*_*j*_*V*_*j *_= *U*_*j*_*S*_*j *_contains the projections of the rows (genes) of *X*_*j *_onto the basis *V*_*j*_. The entries of *S*_*j *_(singular values) give the relative importance of the rows of *V*_*j*_. If the first entry of *S*_*j *_is much larger than the second entry then we know that most of the information in the rows of *X*_*j *_is captured by a single dimension. We thus define the internal consistency of the *j*th cluster to be the ratio of the first and second singular values in *S*_*j*_. The internal consistency provides a measure of how well a single dimension can describe all genes. We can evaluate the quality of a clustering with *K *clusters by the average internal consistency (AICo) of the *K *clusters. The high value of the AICo indicates the good quality of the clusters

## Results

This section uses a synthetic and two real-life gene expression datasets to investigate the performance of the GWKMA in terns of AARI and AICo, while compared with the widely used k-means.

### Synthetic dataset (SYN)

A synthetic dataset is generated by the sine function modeling cyclic behaviour of genes employed by Yeung, et al. [19]. Let *x*_*ij *_be the simulated expression level of gene *i *and time point *j *in the dataset and be modeled by *x*_*ij *_= *λ*_j _* *φ*(*i*, *j*)(1 + *α*_*ij*_), where *φ*(*i*, *j*) = sin(2*πj*/8 - *w*_*k*(*i*) _+ *ε*_*ij*_). *λ*_*j *_is the amplitude control at time *j*, which is chosen according to the standard normal distribution. *φ*(*i*, *j*) models the cyclic behaviour of genes. Each cycle is assumed to span 12 time points. Different clusters are represented by different phase shifts, and *w*_*k*(*i*) _represents a phase shift for gene *i *in cluster *k*, which is chosen according to the uniform distribution on interval [0, 2*π*]. The random variable *ε*_*ij *_represents the noise of gene synchronization, which is chosen according to the normal distribution with the mean of zero and the standard deviation of 0.3. *α*_*ij *_represents the error of gene *i *at time *j*, which is chosen according to the normal distribution with the mean of zero and the standard deviation of 0.4. Using the model above, a synthetic dataset is generated consisting of expression levels of 600 genes at 12 time points. These 600 genes belong to six clusters, each of which contains 100 genes.

### Two real-life datasets

The first real-life dataset is a subset of gene expression profiles over 11 time points collected during the process of bacterial cell division [20], and contain 431 gene expression profiles with the standard deviation greater than 0.5 and no missing data points, denoted by BAC in this paper. The second dataset is a subset of gene expression profiles over 7 time points collected during the developmental program of sporulation in budding [21], and contains 529 gene expression profiles with the standard deviation greater than 1.0, and no missing data points, denoted by SPO. These two original datasets are publicly available from the Stanford microarray database [22] at .

In the experiments conducted in this study, the number of generations is set to be *GEN *= 15, the population size = 21, and the mutation probability *Pm *= 0.10. The Matlab™ software package was used to conduct our experiments. Both AARI and AICo are computed over a variety of the numbers of clusters and for a number of the running results of both GWKMA and the traditional k-means.

**Figure 3:**
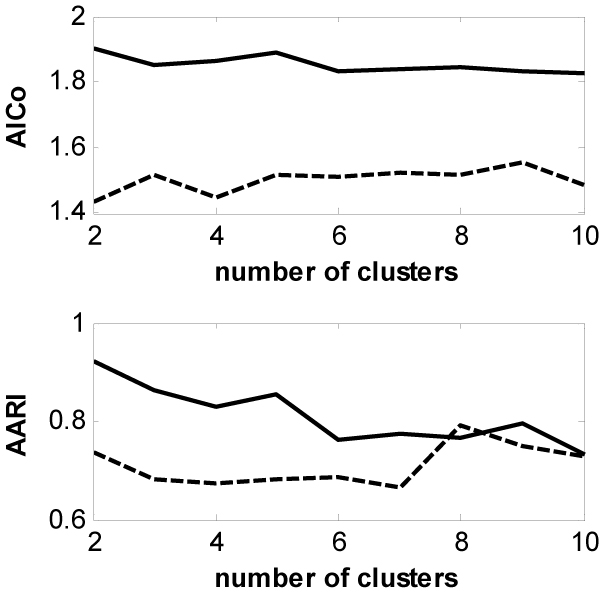
Comparison of the GWKMA (solid lines) to the k-means (dash lines) over a variety of the numbers of cluster on dataset SYN.

The AICos with the cluster numbers from 2 to 10 are calculated from the results of 5 runs of both the GWKMA (solid lines) and the k-means (dash lines), and are depicted in the upper panel of Figure 3. The values of AICo for the GWKMA are greater than 1.8 while those for the k-means are less than 1.6. This indicates that the quality of clustering from the GWKMA is higher than that from the k-means. The AARI with the cluster numbers from 2 to 10 are calculated form the results of 5 runs of both the GWKMA (solid lines) and the k-means (dash lines), and are depicted in the lower panel of Figure 3. The values of AARI for the GWKMA are greater than those for the k-means over all the clusterings except for the one with *k *= 8. This result means that the GWKMA is more insensitive to initial partitions than the k-means.

**Figure 4:**
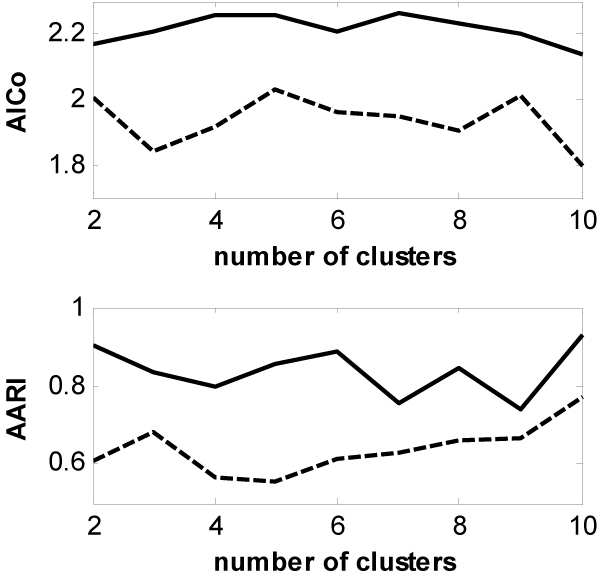
Comparison of the GWKMA (solid lines) to the k-means (dash lines) over a variety of the numbers of cluster on dataset BAC.

**Figure 5:**
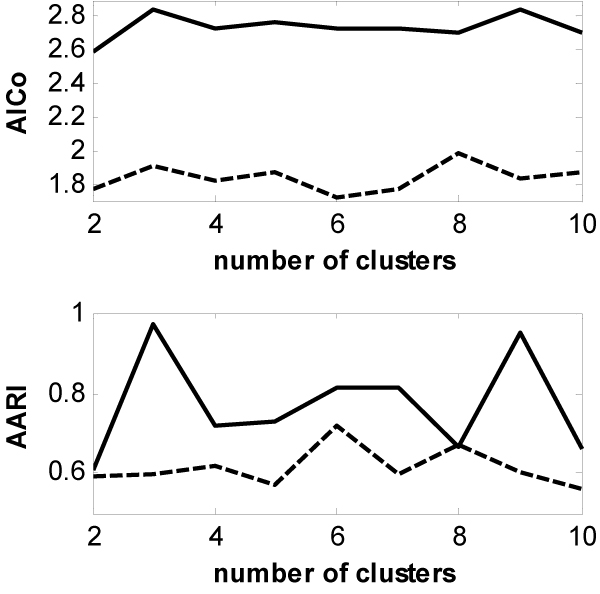
Comparison of the GWKMA (solid lines) to the k-means (dash lines) over a variety of the numbers of cluster on dataset SPO.

Figures 4 and 5 depict the comparisons of the GWKMA and the k-means in terms of the AICo (the upper panels) and AARI (the lower panels) for the two real-life gene expression datasets. Before the clustering, two datasets are normalized by shifting the median of each gene expression profile to zero. From Figures 4 and 5, the same results are obtained from the real-life gene expression data as those from the synthetic dataset. That is, the GWKMA is better than the k-means in terns of AARI and AICo.

The superior quality of clustering from the GWKMA can be explained as follows. The k-means method assumes that 1) all attributes (data at time points) of objects (genes) are independent and 2) the standard deviations of all attributes over all objects are equal.

**Figure 6:**
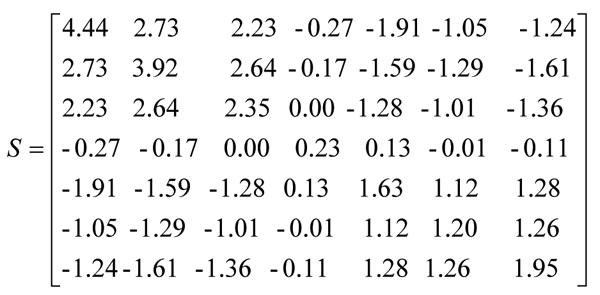
The sample covariance matrix of dataset SPO.

In practice, these two assumptions are not true. For example, we calculate the sample covariance matrix of dataset SPO shown in the matrix S in Figure 6. The elements on the main diagonal of matrix S are not equal and instead range from 0.23 to 4.44. This indicates that assumption 2) for the k-means is invalid. Actually, in many data analysis cases, gene expression data is normalized such that the standard deviation of each attribute over all objects is 1. In this case assumption 2) for the k-means is valid. However, assumption 1) for the k-means is still invalid. For example, in the matrix S in Figure 6 most off-diagonal elements are far from zero. This means most attributes in dataset SPO are correlated and thus not independent.

## Conclusion

In this study, a genetic weighted k-means algorithm (GWKMA) is proposed which is a hybrid algorithm of the weighted k-means algorithm and a genetic algorithm. GWKMA was run on one synthetic and two real-life gene expression datasets. The results of the computational experiments show that the GWKMA performs better than the k-means in terms of the cluster quality (AARI) and the clustering sensitivity to initial partitions (AICo).

In real-life datasets, the assumptions for the k-means are typically not satisfied. The weighted k-means does not needs the assumptions for the k-means. However, like the k-means, the weighted k-means is also sensitive to initial partitions. The proposed GWKMA possesses the merits of both genetic algorithm and the weighted k-mean algorithm, and thus overcomes the disadvantages of the k-means and the weighted k-means. In addition, the proposed algorithm is generic and could have applications to clustering large-scale biological data such as gene expression data and peptide mass spectral data.

## Competing interests

The author declares that they have no competing interests.
